# Finite-size effect on the dynamic and sensing performances of graphene resonators: the role of edge stress

**DOI:** 10.3762/bjnano.7.61

**Published:** 2016-05-09

**Authors:** Chang-Wan Kim, Mai Duc Dai, Kilho Eom

**Affiliations:** 1 School of Mechanical Engineering, Konkuk University, Seoul 05029, Republic of Korea; 2 Biomechanics Laboratory, College of Sport Science, Sungkyunkwan University (SKKU), Suwon 16419, Republic of Korea

**Keywords:** edge stress, graphene resonator, mass sensing, nonlinear vibration, size effect, sensitivity

## Abstract

We have studied the finite-size effect on the dynamic behavior of graphene resonators and their applications in atomic mass detection using a continuum elastic model such as modified plate theory. In particular, we developed a model based on von Karman plate theory with including the edge stress, which arises from the imbalance between the coordination numbers of bulk atoms and edge atoms of graphene. It is shown that as the size of a graphene resonator decreases, the edge stress depending on the edge structure of a graphene resonator plays a critical role on both its dynamic and sensing performances. We found that the resonance behavior of graphene can be tuned not only through edge stress but also through nonlinear vibration, and that the detection sensitivity of a graphene resonator can be controlled by using the edge stress. Our study sheds light on the important role of the finite-size effect in the effective design of graphene resonators for their mass sensing applications.

## Introduction

Recent advances in nanotechnology have allowed for the development of nano-electro-mechanical system (NEMS) devices that can perform mechanical and/or electrical functions [1–3]. In recent years, among NEMS devices, a nanomechanical resonator has received significant attention due to its ability to exhibit unprecedented dynamic frequency ranges even up to gigahertz regime [4–6]. This unprecendented dynamic frequency range of nanomechanical resonator is attributed to the fact that the scaling down of a mechanical resonator leads to an increase of its resonant frequency. Specifically, the resonant frequency (ω) of a nanomechanical device is inversely proportional to the square root of its length (*L*), i.e., 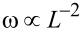. The high-frequency dynamic range of nanomechanical resonators has enabled them to be used for development of lab-on-a-chip mass spectrometry [7–8].

For the recent decade, graphene, which is an atomically thin sheet made of carbon atoms, has been highlighted due to its excellent electrical [9–11] and mechanical [12–13] properties, which imply that graphene can be used for developing a multifunctional NEMS device. For instance, a monolayer graphene sheet has recently been reported to exhibit a high elastic modulus of the order of 1 TPa [14–16], which is much larger than that of conventional engineering materials such as high-tensile steel. This indicates that graphene can be an excellent candidate for developing nanomechanical devices. In recent years, researchers at Cornell [17] have first reported the development of graphene-based nanomechanical resonators, whose dynamic frequency range is of the order of 1 MHz to 1 GHz [18–21]. This high-frequency dynamics of a graphene resonator is ascribed to its remarkable elastic modulus and its low mass density. Here, the resonant frequency of a graphene operating in the harmonic oscillation regime is given by a relation of 
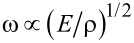
 with *E* and ρ being the elastic modulus and mass density of a graphene, respectively. Since the pioneering work by researchers at Cornell [17], there have recently been efforts to develop the graphene-based resonators for applications in actuation and sensing (e.g., atomic mass detection). The high-frequency dynamic range of graphene resonators has led researchers to develop a graphene-based mass sensor that is able to measure minute amounts of atoms or molecules [19,22–24]. Specifically, the adsorption of a few atoms or molecules can induce an experimentally measurable change in the resonant frequency of the graphene sheet [19].

To improve the dynamic and sensing performance of a graphene resonator, it is necessary to decrease its size, since the miniaturization of a nanomechanical resonator increases its frequency dynamics and sensing performance [4,25]. When the size of a graphene sheet is reduced to the nanometer scale, the edge atoms, which have different bond coordination numbers than bulk atoms, begin to play a vital role in the dynamic behavior of graphene. In particular, this imbalance between the coordination numbers of edge atoms and bulk atoms gives rise to edge stress on a graphene sheet [26–27]. This edge stress is conceptually equivalent to the surface stress that appears in one-dimensional nanostructures such as nanowires [4,26,28]. Recently, Shenoy and coworkers [26–27] have shown that edge stress plays a crucial role in the wrinkling behavior of graphene, and that the elastic properties of graphene are affected by the edge stress. Park and a coworker [29] investigated the role of edge stress on the dynamic behavior (e.g., Q-factor) of a graphene resonator by using atomistic simulations. Despite these previous studies [26–27,29], it has not been fully understood yet how the edge stress has an impact on the dynamic and sensing performances of a graphene resonator. The effect of edge stress on the nonlinear vibration of a graphene resonator has not been studied, even though the graphene resonator can easily reach the nonlinear vibration regime due to the fact that the vibration-driven deflection of a graphene resonator is much larger than its thickness [19–20,30]. In addition, it has not been fully investigated yet how the sensing performance of a graphene resonator can be affected by the edge stress.

In this work, we have studied the role that edge stress plays in the dynamic behavior of a graphene resonator and its sensing performance by using a continuum elastic model, which was developed based on von Karman plate theory [31] with including the effect of edge stress. In particular, we modified the plate theory in order to include the effect of edge stress based on an energetic model as has been also described in surface elasticity theory [4,27]. It was found that edge stress has an impact on both the harmonic and nonlinear oscillations of a graphene resonator, and that the detection sensitivity of a graphene resonator depends on the edge stress. Our study sheds light on the important role of edge stress in designing a graphene resonator for further applications in sensing and actuation.

## Theory and Model

### Effect of edge stress on the mechanics of graphene

The potential energy of a vibrating graphene sheet is composed of bulk strain energy and edge strain energy, conceptually similar to the principle of the surface Cauchy–Born rule [4,32–33].

[1]
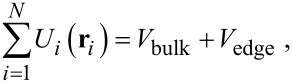


where *U*(**r***_i_*) is the potential energy described to an atom *i*, *V*_bulk_ is the bulk strain energy, and *V*_edge_ is the edge strain energy. Here, the bulk strain energy (*V*_bulk_) consists of bending energy (*V*_B_), twisting energy (*V*_T_), and stretching energy (*V*_S_), which are given by [30–31]

[2]
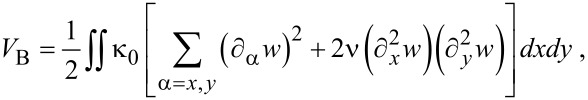


[3]
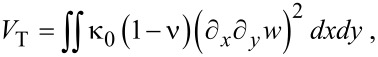


[4]
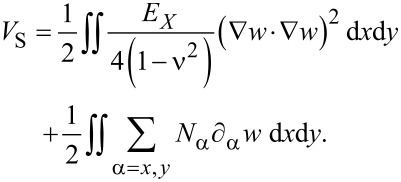


where *w* is the transverse deflection of a graphene, κ_0_, *ν*, and *E_X_* represent the bending rigidity, Poisson’s ratio, and stretching rigidity of the graphene, respectively, *N*_α_ is an axial force that stretches the graphene, and the index α indicates the coordinate *x* or *y*. Here, the coordinate (*x*, *y*) is defined along the edge of the graphene sheet.

Based on the edge energy given by Equation 1, the edge energy density ψ_edge_, which is defined as the edge energy per the unit area of graphene (i.e., *V*_edge_ = ∫∫ψ_edge_ d*x*d*y*), is represented in the form of [27,34–36]


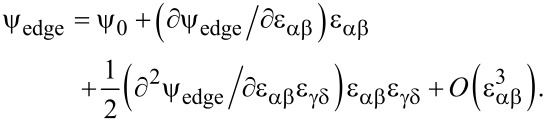


From the edge energy density, the edge stresses, namely constant edge stress (τ^0^) and strain-dependent edge stress (referred to as edge elastic modulus, *Y*), are defined as follows:

[5]
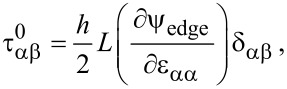


[6]
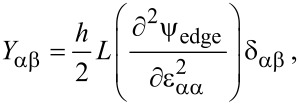


where *L* and *h* are the length and thickness of a square graphene sheet, and δ_αβ_ is the Kronecker delta. Here, a repeated Greek index does not indicate the Einstein summation. In this work, we assume a square graphene sheet, unless otherwise specified. The constant edge stress and edge elastic modulus of a graphene sheet with its specific edge structure can be computed from atomistic simulations [27]. The values of edge stress and edge elastic modulus are summarized in Table 1.

**Table 1:** Constant edge stress and edge elastic modulus for graphene sheets with their various edge structures [27].

edge structures	constant edge stress (nN)	edge elastic modulus (nN)

zigzag (ZZ)	23.577	−3.279
armchair (AC)	18.045	−1.689
ZZ terminated with hydrogen (ZZH)	−6.514	−0.368
AC terminated with hydrogen (ACH)	−47.134	3.912
ZZ edges reconstructed with pentagons and heptagons (ZZ57)	27.189	−2.460
AC edges reconstructed with pentagons and hexagons (AC56)	56.615	0.0038

To gain insight into the role of edge stresses on the mechanics of graphene, we consider graphene modeled as a thin plate, which is deformed due to the sole bending of graphene with only a bending strain ε*_xx_* given as ε*_xx_* = *C_x_z*, where *C_x_* is the bending curvature in the *x*-direction, and *z* is a coordinate defined along the thickness of the graphene sheet. At this point, for the sake of convenience, it is assumed that the nonlinear bending deformation of graphene is neglected (i.e., small deformation), and that no axial force *N*_α_ is applied to the graphene sheet. For each edge, the edge stress acting on the graphene sheet is given as [34–36]

[7]
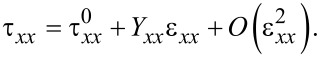


Here, it should be noted that the edge stress given by Equation 7 is conceptually equivalent to the surface stress appearing in a three-dimensional crystal [34–36]. The edge stress generates the residue stress (*R_xx_*) in order to satisfy Newton’s third law as follows:

[8]
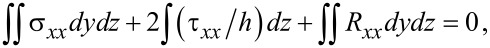


where *σ_xx_* is the bending stress acting on graphene given by

[9]
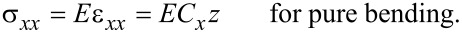


Here, *E* is the bending elastic modulus of graphene. Because of the symmetry of bending stress with respect to the *z*-axis, Newton’s third law depicted in Equation 8 can be reduced into the following form

[10]
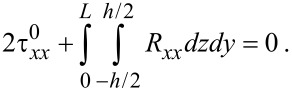


The bending moment per unit length of graphene can be obtained from

[11]
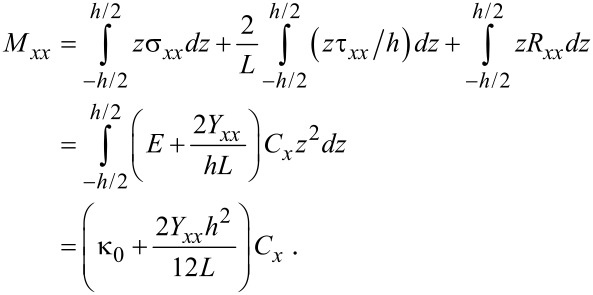


Here, κ_0_ is the bending rigidity of graphene defined as κ_0_ = ∫*Ez*^2^d*z*. As shown in Equation 11, the constant edge stress (τ^0^) does not contribute to the bending deformation of graphene, while the bending mechanics of graphene are critically affected by the edge elastic modulus.

In general, when graphene is deformed by an in-plane bending strain [31], the bending moments acting on a graphene sheet are represented in the following form.

[12]
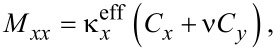


[13]
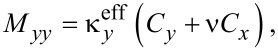


where 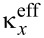 and 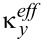 are the effective bending rigidities defined as

[14]
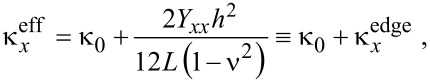


[15]
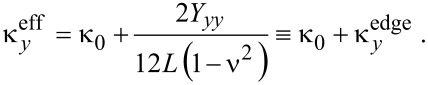


Here, κ_0_ is the bending rigidity of graphene given by κ_0_ = *Eh*^3^/12 (1 − ν^2^) [31]. Based on bending moments given by Equation 12 and Equation 13, as well as on von Karman plate theory, the force acting on the unit area of a graphene is

[16]
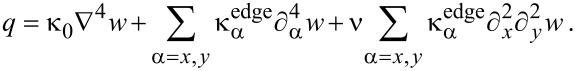


The equation of motion for a graphene sheet, which is deformed by only an in-plane bending strain without any applied stretching force as well as without any nonlinear effect (i.e., small deformation), is given by Equation 17 where ρ is the mass per unit area of graphene. The first term in Equation 17 indicates the inertia force acting on graphene.

[17]
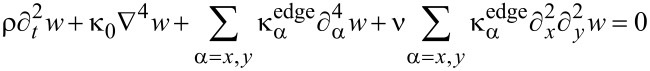


In general, when a graphene resonator is actuated by an electrostatic force *f*(*x*,*y*,*t*) and a stretching force is applied to the graphene sheet, the equation of motion for the graphene sheet, assuming large deformation (i.e., including the nonlinear effect), is represented in the form of Equation 18.

[18]
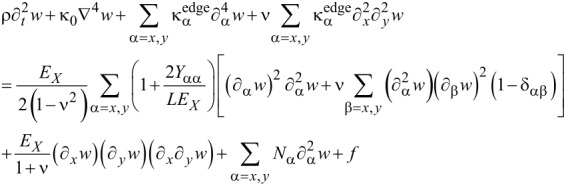


Here, we note that the large deformation of a graphene resonator is attributed to the fact that the transverse deflection of a graphene resonator during its vibration is much larger than the thickness of the graphene.

### Equation of motion: Duffing equation

In order to understand the dynamic behavior of a graphene resonator, we need to numerically solve the equation of motion given by Equation 18, which is not straightforward. To numerically treat the equation of motion, we employed Galerkin’s method [37–38], where the transverse deflection of graphene is assumed to be in the form of 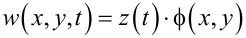 with *z*(*t*) and 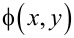 being the time-dependent amplitude of transverse deflection and the deflection eigenmode, respectively. Consequently, the equation of motion can be written as shown in Equation 19.

[19]
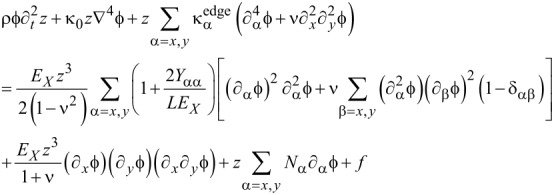


By multiplying the deflection eigenmode 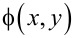 into Equation 19 followed by the integration by parts, the equation of motion becomes the Duffing equation [39–41] as follows:

[20]
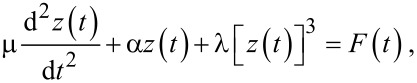


where μ, α, λ and F(t) are given by Equation 21, Equation 22, Equation 23 and Equation 24, respectively.

[21]
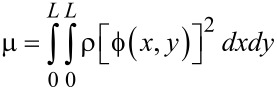


[22]
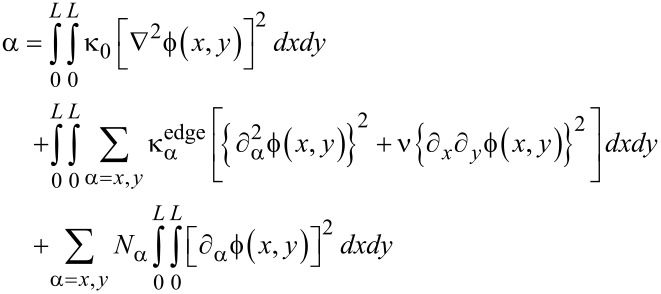


[23]
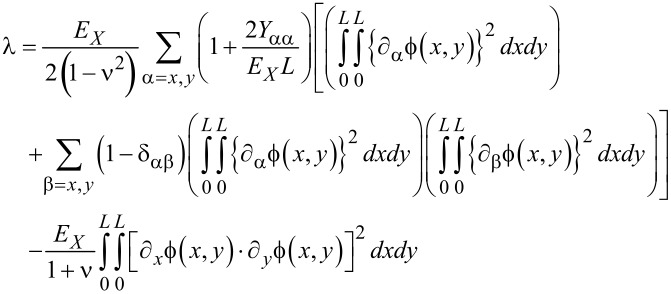


[24]
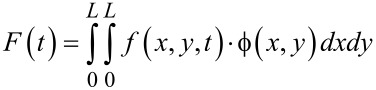


When a graphene resonator is actuated by an ac voltage, the electrostatic force *f*(*x*,*y*,*t*) can be written as *f*(*x*,*y*,*t*) = *g*(*x*,*y*)·cos Ω*t*, where Ω is the driving frequency. Then, the driving force *F*(*t*) in the Duffing equation becomes *F*(*t*) = *p*_0_·cos Ω*t*, where 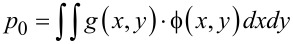. Here, it should be noted that the cofficients of the Duffing equation are computed based on the deflection eigenmode given by 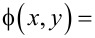 (2/3)[1 − cos(2π*x*/*L*)][1 − cos(2π*y*/*L*)], which satisfies the essential boundary conditions [22].

For the case in which atoms are adsorbed onto a graphene resonator, we need to update the coefficient μ, while the other parameters of the Duffing equation are not affected by atomic adsorption, since the atomic adsorption only affects the inertia term dictated by the coefficient μ. When atoms are adsorbed onto a specific graphene site, the coordinates of which are given by (*x_m_*, *y_m_*), the inertia becomes

[25]
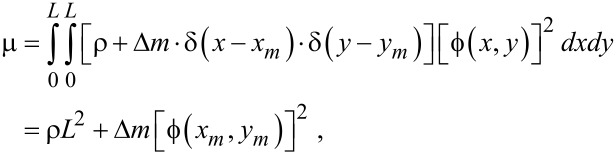


where Δ*m* is the total mass of atoms adsorbed onto the specific site of a graphene, and δ(*x*) is the Dirac delta function. For the case in which atoms are adsorbed uniformly onto the entire area of a graphene sheet, the inertia term can be written as

[26]
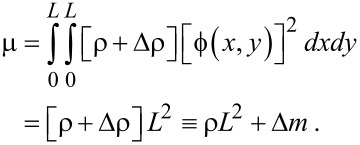


Here, Δρ is the mass per unit length for adsorbed atoms. It is straightforward to compute the frequency shift (Δω) of a graphene resonator due to atomic adsorption such as Δω = ω(*m* + Δ*m*) − ω(*m*), where ω(*m* + Δ*m*) is the resonant frequency of a graphene resonator onto which atoms are adsorbed, and ω(*m*) is the resonant frequency of a bare graphene resonator. Here, we note that since the elastic modulus of adsorbed atoms is much smaller than that of the graphene resonator, the frequency shift of the graphene resonator is mostly attributed to the mass of adsorbed atoms [42]. If the elastic modulus of adsorbed molecules is comparable to that of a resonator, then the molecular adsorption may increases the frequency of the resonator [43–44], which is not relevant for the case of atomic adsorption onto a graphene resonator.

## Results and Discussion

### The effect of edge stress on the harmonic oscillation of a graphene resonator

In order to validate our model, we computed the effective elastic modulus of a graphene sheet as a function of its size based on the harmonic oscillation of graphene. Specifically, we consider the vibration of a graphene sheet actuated by a force of *p*_0_ = 0.001 aN. This actuation force is sufficient to guarantee the harmonic oscillation of graphene [22]. The relationship between the effective elastic modulus (*E*_eff_) of graphene and its resonant frequency (ω_0_) is given as [18,45]

**Figure 1:**
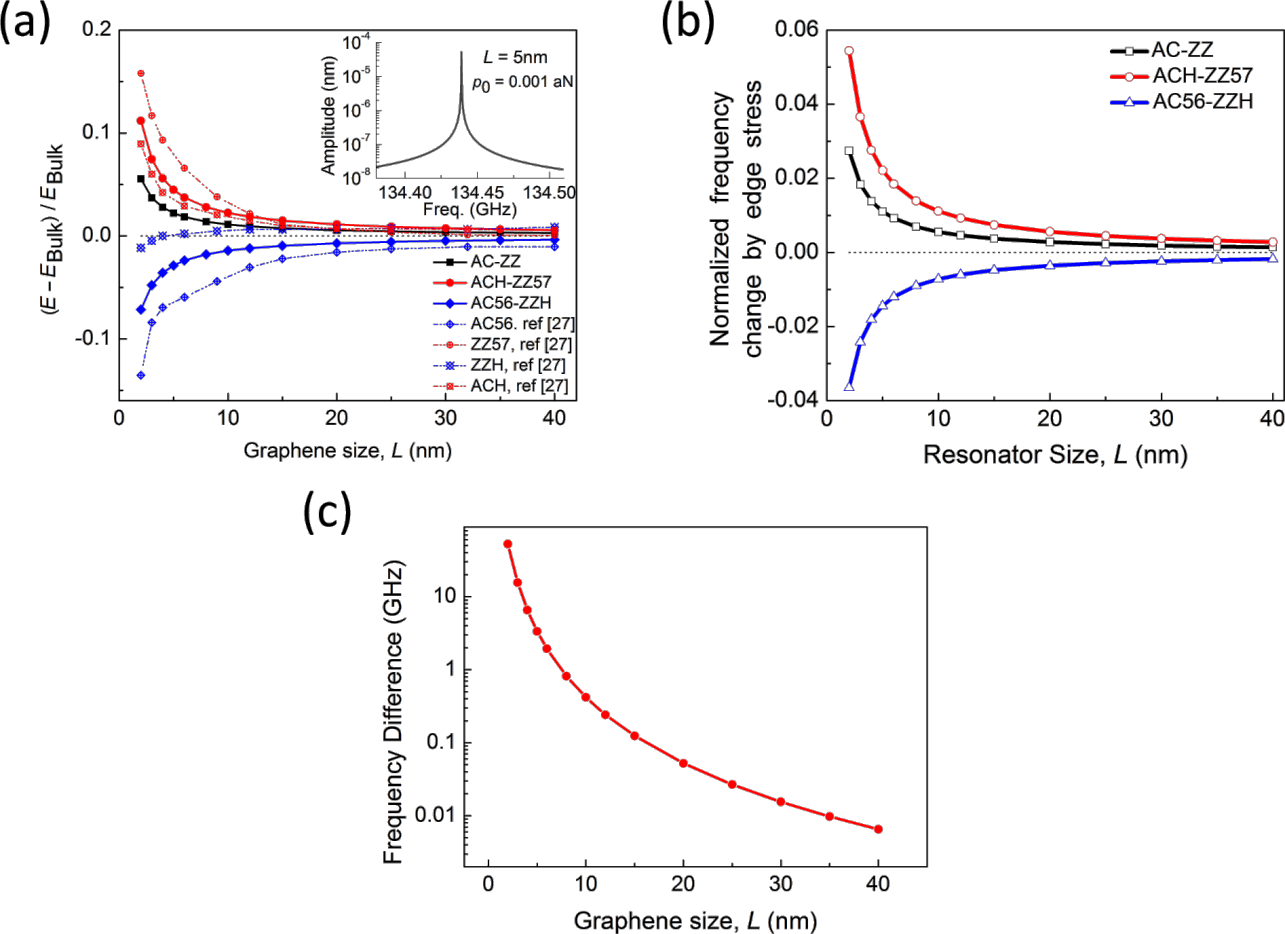
(a) Effective elastic moduli of graphene sheets as a function of their sizes and edge structures. Here, the effective elastic moduli were obtained based on the relation given in Equation 27. (b) Normalized frequency change of graphene due to edge stress. (c) Differences between resonant frequencies of graphene resonators with edge structures of ACH-ZZ57 and AC56-ZZH. When the size of the graphene resonators is below 10 nm, the frequency difference between these two is calculated to be above 1 GHz.

[27]
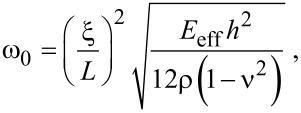


where *E*_eff_, *h*, *L*, ρ, and ν are the effective elastic modulus, thickness, length, mass density, and Poisson’s ratio of graphene, respectively, and ξ is a constant eigenvalue depending on the boundary conditions. Figure 1a shows a dimensionless quantity χ = (*E*_eff_ – *E*_bulk_)/*E*_bulk_, where *E*_bulk_ is the elastic modulus of an infinitely long graphene sheet, as a function of the size of graphene sheets and their various edge structures. This dimensionless quantity shows how the edge stresses affect the elastic modulus of graphene. When the size of the graphene sheet with edge structures of AC-ZZ or ACH-ZZ57 decreases, the elastic modulus increases. This is attributed to the positive elastic moduli (*Y_xx_* and *Y_yy_*) of the edges. On the other hand, when the size of an AC56-ZZH graphene sheet decreases, its elastic modulus does as well, because of the negative elastic moduli of the edge. In addition, it is found that the edge eleasic modulus plays a crucial role in the elastic properties of graphene sheets when their size is *L* < 25 nm (Figure 1a). The values of effective elastic moduli of graphene, which were obtained from the resonant frequencies in this work, are quantitatively comparable to those predicted from atomistic model-based thermodynamics theory [27]. The discrepancy between results obtained from our model and thermodynamics model [27] is ascribed to the fact that the thermodynamic model in [27] assumes two infinite edges and two finite edges, while our model considers four finite edges.

Now, we investigate the role that the edge stresses play in the harmonic oscillation behavior of a graphene resonator. Here, we define the frequency shift caused by edge stress as Δω_edge_ = ω_edge_ − ω_0_, where ω_edge_ is the resonant frequency of graphene measured from the modified plate theory (i.e., including edge stress effects). It is shown in Figure 1b that the decrease of the length of a graphene with AC-ZZ or ACH-ZZ57 edge structure results in an increase in the resonant frequency. Contrarily, when the length of an AC56-ZZH graphene sheet decreases, the resonant frequency also decreases. It should be noted that the constant edge stress does not affect the resonant frequency of graphene (Equation 11). This is similar to the case of nanowire resonators, the frequency dynamics of which are not affected by the constant surface stress [28]. The frequency shift due to edge stress can be analytically understood by using the modified plate theory. Specifically, based on Equation 14 and Equation 15, the effective bending rigidity (κ_eff_) of graphene can be asymptotically given by

[28]
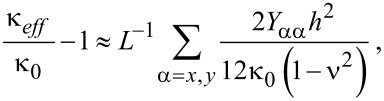


where κ_0_ is the bending rigidity of infinitely long graphene (i.e., without any edge stresses). As a consequence, the frequency change of graphene due to edge stress is represented in the form of

[29]
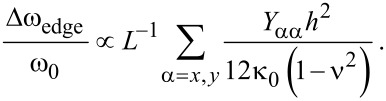


The scaling law of the frequency shift due to edge stress elucidates that the frequency shift due to edge stress increases with decreasing the size of graphene. This theoretical prediction is consistent with our simulation results (Figure 1b), which shows that the frequency shift of graphene due to edge stress is inversely proportional to the length scale of the graphene resonator.

To quantitatively understand how the edge stress affects the dynamic behavior of a graphene resonator, we consider the resonant frequencies of two types of graphene resonators, the edge structures of which are given as AC-ZZ and AC56-ZZH. We measured the frequency differences between these two graphene resonators as a function of their length scales. Figure 1c shows that the frequency difference between these two graphene resonators becomes large even up to the order of 500 MHz to 10 GHz when their size is below 10 nm. This observation suggests that the edge stress is a key parameter that governs the dynamic behavior of graphene resonators whose size (i.e., length) is restricted below 25 nm (Figure 1c). For example, in case of a length of 40 nm, the frequency difference between two aforementioned graphene resonators is calculated to be ca. 6 MHz, which is by three orders of magnitude smaller than the frequency difference for a length below 10 nm.

### Edge stress effect on the nonlinear vibration of graphene

We have studied the effect of edge stress on the nonlinear vibration of a graphene resonator. Nonlinear deformation can easily occur because the transverse deflection of a graphene sheet during its vibration is typically much larger than the thickness of the sheet. In addition, as described in [22–24,26], the nonlinear vibration is an effective route to improve the dynamic performances of NEMS resonators. To the best of our knowledge, the finite-size effect (i.e., edge stress effect) on the nonlinear vibration of a graphene resonator has not been systematically studied yet.

**Figure 2:**
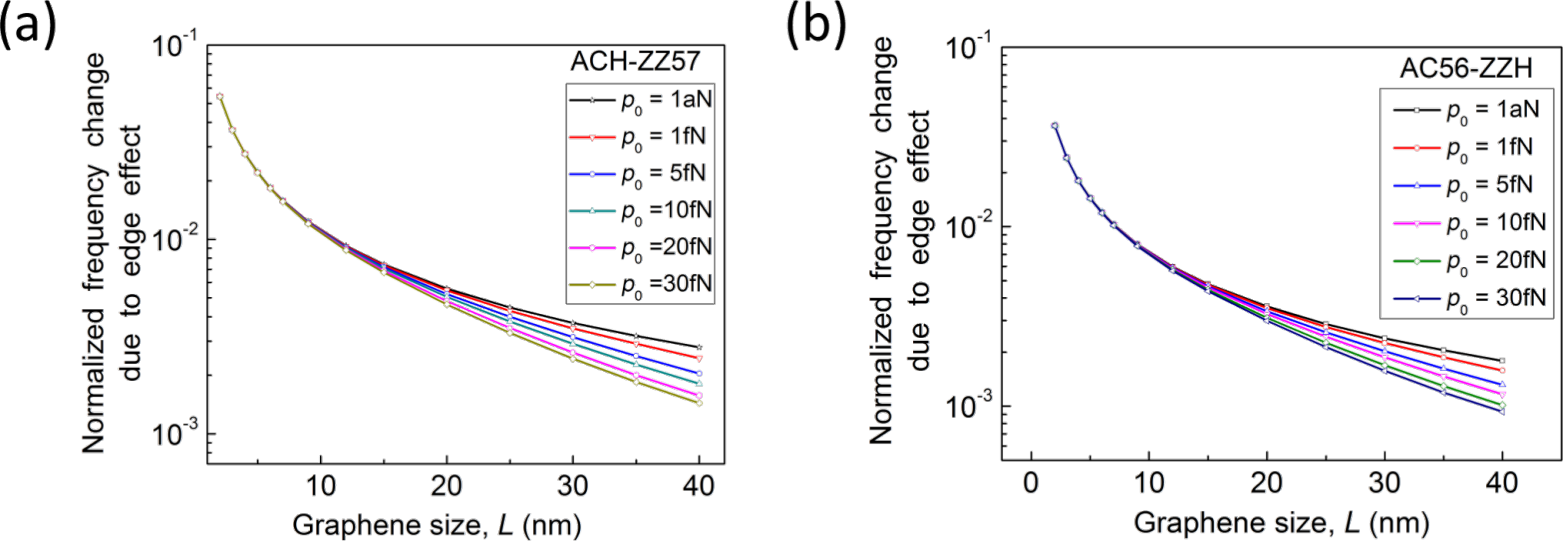
Normalized frequency shift due to edge stress for a graphene sheet with (a) ACH-ZZ57 and (b) AC56-ZZH edge structure as a function of the size of the graphene resonator and of the actuation force.

We consider the vibration of a graphene sheet actuated by a force of *p*_0_ > 1 fN, at which the nonlinear vibration of graphene can be observed [22]. It occurs because of the geometric nonlinear effect, which arises from the clamped boundary conditions. It is shown in Figure 2 that the frequency shift due to edge stress is critically dependent on the actuation force *p*_0_ when the size of the graphene becomes *L* > 20 nm. The frequency shift of a small-scale graphene sheet (*L* < 20 nm) due to edge stress exhibits no nonlinear behavior. Moreover, we found that as the actuation force *p*_0_ increases, the frequency shift of a large-scale graphene resonator (*L* > 20 nm) due to edge stress is decreasing as the actuation force increases. This indicates that nonlinear vibration can reduce the effect of edge stress on the frequency dynamics of a graphene resonator. These observations indicate that the dependence of the frequency dynamics of graphene resonators on the edge stress is determined by two physical parameters such as the graphene size, *L*, and actuation force, *p*_0_. In particular, in case of *L* > 20 nm and *p*_0_ > 1 fN (i.e., when the small-scale graphene resonator operates in the nonlinear vibration regime), the edge stress does not have any effect on the frequency dynamics of the graphene resonator.

This effect of nonlinear vibration on the edge stress-dependent frequency dynamics of a graphene resonator can be elucidated with using our modified plate theory (Equation 20). The nonlinearity of a vibrating graphene sheet can be defined as [39]

[30]
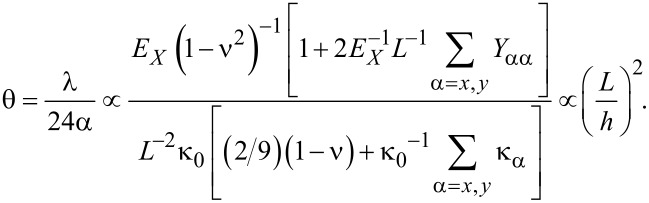


The scaling law for the nonlinearity given in Equation 30 demonstrates that the nonlinearity of a vibrating graphene sheet depends on its size. The nonlinearity increases with the size of the graphene resonator. When the size of graphene becomes smaller than 20 nm, the frequency dynamics of graphene becomes independent of the actuation force *p*_0_, which is attributed to the edge elastic modulus that can annihilate the nonlinear effect. This theoretical prediction is consistent with the simulation results (Figure 2) showing that the resonant frequency of graphene resonators smaller than 20 nm is independent of the actuation force.

### The influence of edge stress on the sensing performance of graphene resonators

In order to understand how the finite-size effect affects the sensing performance of a graphene resonator , which can serve as a mass sensor [19] capable of measuring the mass of a single or few atoms, we consider the frequency dynamics of a graphene resonator in response to atomic adsorption. It should be noted that the finite-size effect on the sensing performance of a graphene resonator has not been studied yet, though the finite-size effect on the sensing performance of a nanowire resonator [28] was systematically studied. In general, as described in our previous work [22], the frequency shift of a graphene resonator due to atomic adsorption critically depends on the location at which the atoms are adsorbed. We note that the frequency shift of a graphene resonator due to atomic adsorption becomes maximized when few atoms are adsorbed onto the center of a graphene resonator [22].

**Figure 3:**
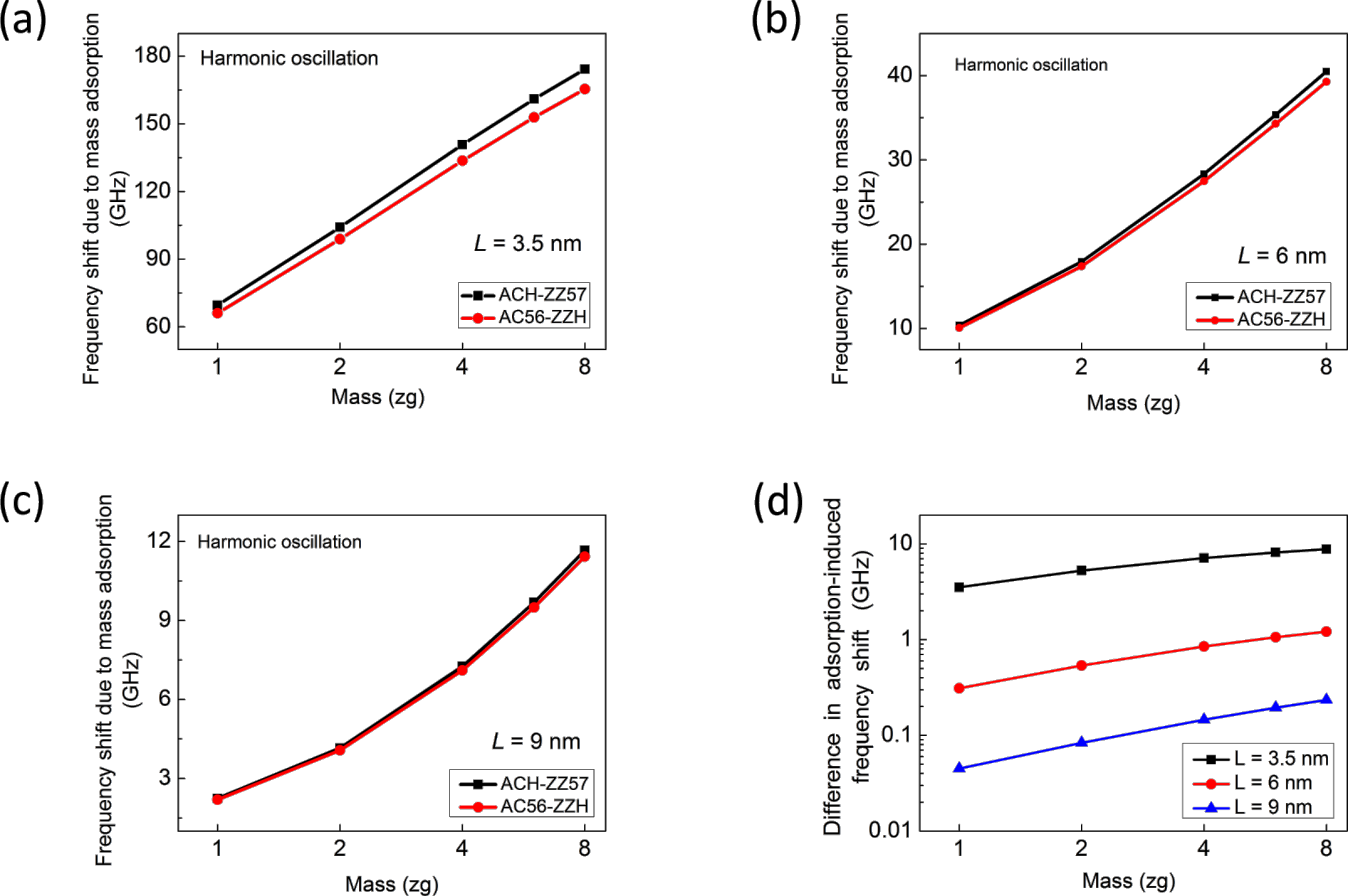
(a–c) Frequency shifts of graphene resonators with ACH-ZZ57 and AC56-ZZH edge structures of as a function of adsorbed mass for a given graphene sheet length of (a) *L* = 3.5 nm, (b) *L* = 6 nm, and (c) *L* = 9 nm, respectively. Here, the frequency shift of the graphene due to mass adsorption was measured based on the harmonic oscillation of the graphene. (d) Difference between the mass-induced shifts of the resonant frequency of the two resonators.

For understanding the role of the edge structure of a graphene resonator in its sensing performance, we study the resonant frequency dynamics for graphene, which operates in harmonic oscillation regime, with ACH-ZZ57 or AC56-ZZH edge structures in response to atomic adsorption. Figure 3a–c shows the frequency shift of graphene resonators with ACH-ZZ57 and AC56-ZZH edge structures due to atomic adsorption as a function of the length of the resonator and the mass of adsorbed atoms, when the graphene resonator is actuated by a force of 0.001 aN. It is found that as the length of a graphene resonator increases, the effect of edge stress on the frequency shift due to atomic adsorption is decreasing. It is shown that a graphene resonator with ACH-ZZ57 edge structure exhibits a better detection sensitivity than a resonator with AC56-ZZH edge structure. This observation suggests that an edge structure that exhibits a negative edge elastic modulus, leads to an increase in the detection sensitivity of a graphene resonator. This edge effect on the detection sensitivity of a graphene resonator disappears when its length becomes larger than 10 nm. To gain a quantitative insight into how the edge effect on the detection sensitivity of a graphene resonator depends on its length, we calculated the difference between mass-induced frequency shifts of two graphene resonators with ACH-ZZ57 and AC56-ZZH edge structures (Figure 3d). We found that when atoms with a total mass of 8 zg are adsorbed onto the center of graphene resonators with a length of 3.5 nm, the difference between mass-induced frequency shifts is calculated to be about 10 GHz. This difference becomes small (ca. 0.1 GHz) when the same amount of atoms is adsorbed onto the graphene sheets with a length of 9 nm.

**Figure 4:**
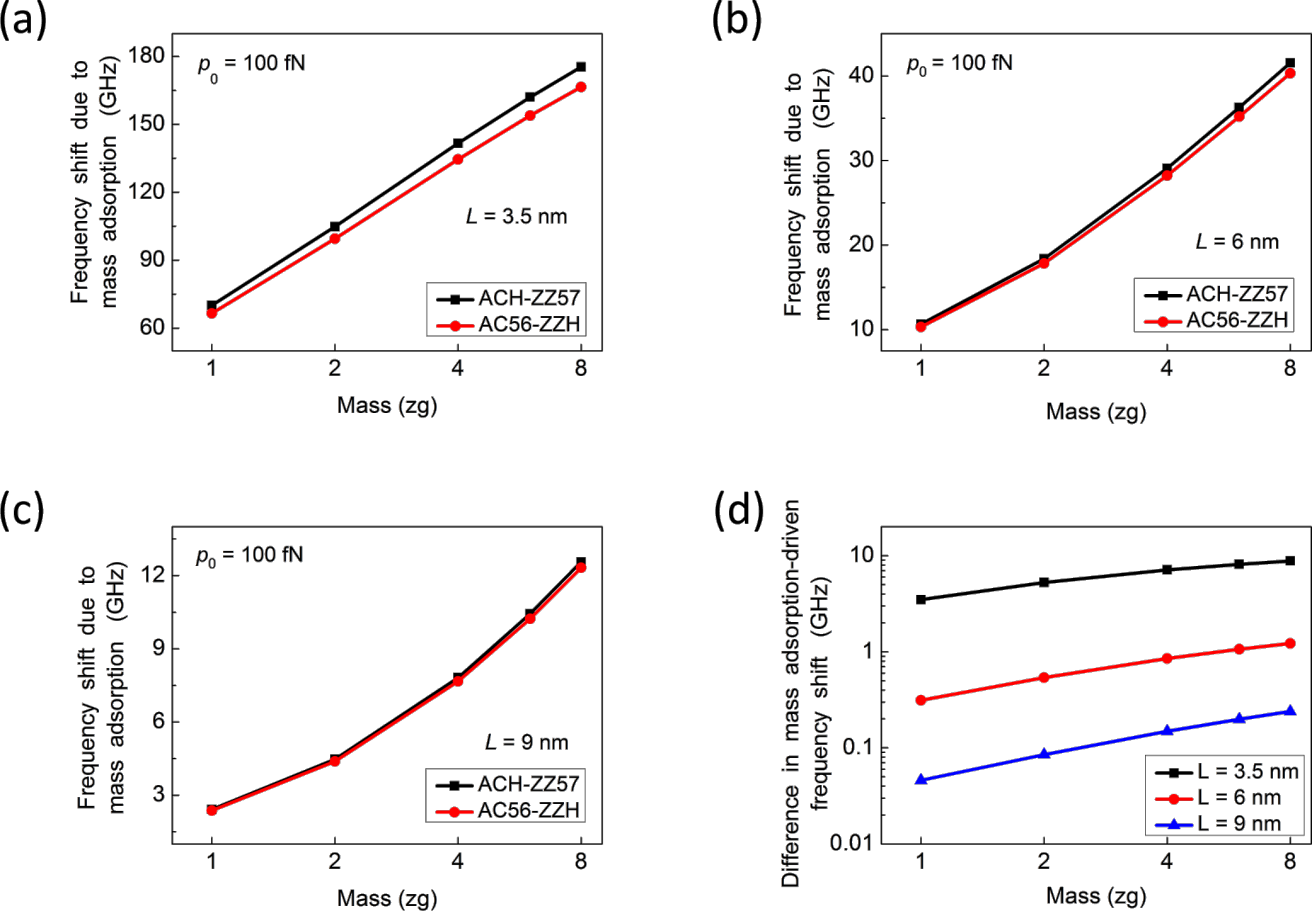
(a–c) Mass adsorption-induced frequency shift of a graphene resonator, with lengths of (a) 3.5 nm, (b) 6 nm, and (c) 9 nm, with either ACH-ZZ57 or AC56-ZZH edge structures. (d) Difference between mass adsorption-induced frequency shifts of the two graphene resonators.

Now, we study the influence of edge stress on the detection sensitivity of a graphene resonator that operates under nonlinear vibration conditions. This is relevant because a graphene resonator can easily undergo the nonlinear vibration due to the large transverse deflection of graphene compared to its thickness [19–20,30]. In addition, nonlinear vibration improves the detection sensitivity of NEMS resonators [22–24,46]. Here, we investigate the detection sensitivity of small-scale graphene resonators under an actuation force of 100 fN. Figure 4a–c depicts the atomic adsorption-induced frequency shift of the graphene resonators with lengths of 3.5, 6, and 9 nm. The edge structures are ACH-ZZ57 or AC56-ZZH. Similar to the case of harmonic oscillations, as the size of a graphene resonator decreases, the edge structure of graphene begins to play a crucial role on its detection sensitivity (Figure 4d). Furthermore, it is found that the detection sensitivity of a graphene resonator with ACH-ZZ57 edge structure is higher than that of a resonator with AC56-ZZH edge structure (Figure 4a–c). We calculated the difference between mass adsorption-induced frequency shifts of these graphene resonators. The effect of the edge structure becomes dominant when the length of the graphene sheet is about 3 nm. It is shown that, for a graphene resonator with a length of less than 10 nm, the increase of actuation force does not improve the detection sensitivity of the graphene resonator. The detection sensitivity is almost independent of actuation force. This can be elucidated from the scaling law given by Equation 30, which shows that the nonlinearity of vibration for a graphene resonator appears as its length increases, while this nonlinearity is likely to disappear when the length of the resonator decreases.

**Figure 5:**
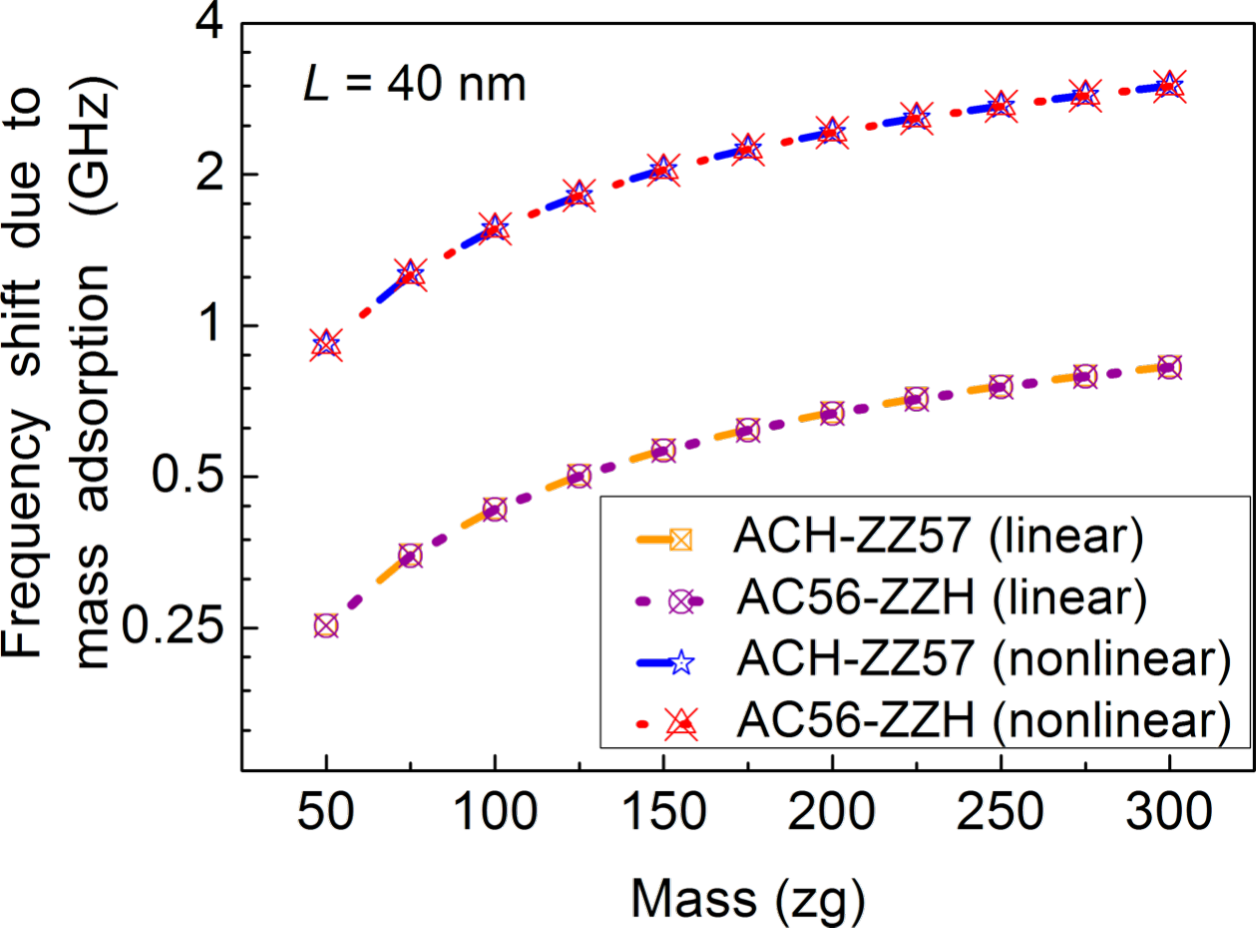
The frequency shift of a graphene resonator (*L* = 40 nm) due to atomic adsorption as a function of the total mass of adsorbed atoms as well as the type of edge structures.

We proceed to characterize the frequency response of two graphene resonators with length *L* = 40 nm and ACH-ZZ57 or AC56-ZZH edge structure. The consideration of a relatively large graphene resonator is attributed to the result from Equation 30 that a larger graphene resonator is likely to more easily undergo nonlinear vibration, and to our previous findings [22,46] that nonlinear vibration improves the detection sensitivity of NEMS resonators. Figure 5 shows that the nonlinear vibration increases the mass adsorption-induced frequency shift of a graphene resonator, while the edge structure does influence the detection sensitivity of the resonator.

To summarize, based on the results shown in Figures 3–5, the detection sensitivity of a graphene resonator can be enhanced through either scaling down or through increasing the actuation force. However, when the down-scaling down of the graphene resonator is considered to improve the detection sensitivity of the graphene, an increase of the actuation force (nonlinear vibration) is unable to enhance the detection sensitivity. The improvement of the detection sensitivity through nonlinear vibration is only possible when the length of the resonator is larger than 20 nm.

## Conclusion

In this work, we have studied the finite size effect on the dynamic behavior and the sensing performance of a graphene resonator with using a modified plate theory, which includes the effect of edge stress based on an energetic model. It is found that the edge stress plays an important role in the dynamic behavior and sensing performance of graphene resonators, whose length scale is below 20 nm. Moreover, it is shown that nonlinear vibration does not improve the detection sensitivity of a small-scale graphene resonator (length scale below 10 nm), whereas the edge stress is a key parameter that determines the dynamic behavior and sensing performance of the small-scale graphene resonators. Contrarily, for a large graphene resonator (length scale above 20 nm), the nonlinear vibration has a critical impact on the dynamic behavior and sensing performance, while these two properties are almost independent of the effect of edge stress. It should be noted that this work based on the modified plate theory is restricted to studying only the resonant frequencies of graphene sheets, while this current work may not be applicable for understanding the Q-factor. Our model does not include the intrinsic (flaw) factors, which affect the Q-factor of a graphene resonator. In order to understand the effect of edge stress on the Q-factor of a graphene resonator [29], the theoretical model described in this work has to be modified by including the intrinsic damping factors such as clamping or support loss [47–49] and thermoelastic damping loss [50], which will be considered in our future work. In conclusion, our work sheds light on the influence of edge stress on the resonant frequency and sensing performance of a graphene resonator, which allows for an insight into design rules for the effective development of graphene-based resonators and their applications in atomic mass sensing.
